# Self Injurious Behaviour (SIB) in Patients with Intellectual Disabilities Presented to the Department of Psychiatry and Behavioural Science Allied Hospital II Faisalabad, Pakistan

**DOI:** 10.1192/bjo.2026.11233

**Published:** 2026-06-30

**Authors:** Asadullah Raoufy, Imtiaz Ahmad Dogar, Sammar Fatima, Pavisha Sajid

**Affiliations:** Faisalabad Medical University, Faisalabad, Pakistan

## Abstract

**Aims:**

Self-injurious behaviour (SIB) is a substantial and challenging behavioural issue among individuals with intellectual disabilities (ID), resulting in heightened morbidity, caregiver stress, and healthcare consumption. Data from low- and middle-income nations, such as Pakistan, continue to be scarce,with intellectual disabilities admitted to a tertiary care psychiatric institution in Faisalabad, Pakistan.Aims were to as certain the prevalence, trends and correlating factors of self-injurious conduct in patients.

**Methods:**

This cross-sectional study was performed in the Department of Psychiatry and Behavioural Sciences, Allied Hospital II, Faisalabad, over a duration of six months. A total of 96 patients, aged 8 years and older, diagnosed with intellectual disability under DSM–5 criteria, were recruited by non-probability consecutive sampling. Demographic data, severity of intellectual disability, existence and kind of self-injurious behaviour (SIB), severity, triggers, and interventions were gathered using a structured proforma. We used SPSS version 27 to do the statistical analysis. We used chi-square tests to look at associations, and a p-value of 0.05 or less was considered statistically significant.

**Results:**

35.4%of the subjects engaged in self-injurious conduct. All instances of self-injurious behaviour (SIB) were seen among the 8–20-year age demographic, with a notable prevalence in men (91.2%). SIB was not present in people with mild intellectual disability but had a significant correlation with moderate, severe, and profound disability (p < 0.001). The most common types of SIB were beating oneself, head-banging, and biting oneself. Sensory overload, difficulty with communicating, and behaviour that seeks attention were some of the most common triggers. Despite the clinical burden, most of the people who were impacted did not get structured behavioural or pharmaceutical help.

**Conclusion:**

Self-injurious behaviour is a prevalent and clinically relevant issue among younger individuals with moderate to severe intellectual disability. To have better results for this vulnerable group, it is important to find them early, train caregivers, and make mental health treatments stronger.

